# Antibiotic Resistance & Extended-Spectrum ß-Lactamase Production in Clinical and Non-Clinical Isolates in Tabuk

**DOI:** 10.3390/medsci12030042

**Published:** 2024-08-18

**Authors:** Badriah Alanazi, Ghulam Muhiuddin, Yazeed Albalawi, Khalid Alhazmi, Othman Alzahrani, Marai Alamri, Hisham Alshadfan, Mohammad Zubair

**Affiliations:** 1 Department of Mathematics, Faculty of Science, University of Tabuk, Tabuk 47512, Saudi Arabia; 2 Department of Obstetrics & Gynecology, Faculty of Medicine, University of Tabuk, Tabuk 47512, Saudi Arabia; 3 Department of Pathology, Faculty of Medicine, University of Tabuk, Tabuk 47512, Saudi Arabia; 4 Department of Biology, Faculty of Science, University of Tabuk, Tabuk 47512, Saudi Arabia; 5 Department of Surgery, Faculty of Medicine, University of Tabuk, Tabuk 47512, Saudi Arabia; 6 Department of Clinical Biochemistry, Faculty of Medicine, University of Tabuk, Tabuk 47512, Saudi Arabia; 7 Department of Medical Microbiology, Faculty of Medicine, University of Tabuk, Tabuk 47512, Saudi Arabia

**Keywords:** clinical, non-clinical, ESBL, CTX-M, TEM, SHV, AmpC

## Abstract

The increasing prevalence of antibiotic resistance, driven by the production of extended-spectrum beta-lactamases (ESBLs), presents a critical challenge to current medical treatments, particularly in clinical settings. Understanding the distribution and frequency of ESBL-producing bacteria is essential for developing effective control strategies. This study investigated the antibiotic resistance and extended-spectrum beta-lactamase (ESBL) production in bacterial isolates in clinical and non-clinical (food) specimens in Tabuk, KSA. A total of 57 bacterial isolates were analysed, with *E. coli* and *Pseudomonas* sp. being the most prevalent. High resistance rates were observed, particularly against third-generation cephalosporins in clinical isolates. ESBL screening revealed a significant prevalence in clinical samples (58.3%), with *E. coli* showing the highest positivity. Conversely, only a low percentage of food isolates were ESBL positive. Molecular analysis confirmed the presence of various ESBL genes, with *bla_CTX_*_-M_ being the most frequent, predominantly found in clinical isolates. This study highlights the concerning levels of antibiotic resistance and ESBL production in the region, emphasising the need for effective infection control measures and prudent antibiotic use.

## 1. Introduction

Antibiotic resistance is a critical threat to global public health as one of the top ten health threats facing humanity today. Traditional medical treatments are being impeded by the proliferation of antibiotic-resistant bacteria, leading to longer hospital stays and higher medical costs, as well as rising death rates. ESBLs have the ability to neutralise various beta-lactam agents, including antibiotics like penicillin and cephalosporin [1]. The unexpected ability of ESBLs to inactivate more advanced cephalosporins is significant because these antibiotics were designed to overcome previously established resistance mechanisms. This highlights the rapid evolution of resistance and complicates treatment options, often necessitating the use of last-resort antibiotics like carbapenems, raising concerns about the potential for further resistance development and emphasising the need for ongoing research and effective antimicrobial stewardship.

Expansions of ESBL-producing bacteria continue to occur worldwide, spreading to the community and healthcare facilities. These sophisticated bacteria have the insidious ability to degrade a wide variety of beta-lactam antibiotics, from third-generation cephalosporins to monobactams, making it difficult to fight infections with traditional treatment methods. The spread of ESBL-producing Enterobacteriaceae, led by notorious culprits such as *E. coli* and *Klebsiella pneumoniae*, is feared because of its association with severe and incurable disease and shows its ability to spread resistance genes in mutational cellular genetic elements of the antibody resistance [2].

The city of Tabuk in western Saudi Arabia is a unique centre for exploring the spread and potential of antibiotic resistance. Tabuk faces comparable challenges in controlling the spread of antibiotic-resistant microorganisms as other cities grapple with population growth and advancements in health infrastructure. In recent years, the global rise in antibiotic resistance has been compounded by the emergence of extended-spectrum beta-lactamases (ESBLs), enzymes that grant bacteria resistance to a variety of beta-lactam antibiotics, including penicillins and cephalosporins. ESBLs are primarily produced by Enterobacteriaceae, which has significant implications in regard to the treatment of bacterial infections. According to Shaikh et al. [2], the growing prevalence of these enzymes necessitates new approaches in both epidemiology and treatment strategies to effectively manage and counteract their impact [2]. In the Tabuk region, research conducted by Alqahtani et al. [3] emphasised the presence of plasmid-mediated ESBL-producing Enterobacteriaceae, highlighting the clinical challenges they pose and the need for effective monitoring and control measures in local healthcare settings [3]. Despite substantial efforts to understand and combat antibiotic resistance, there remains a substantial gap in knowledge, particularly regarding the prevalence of ESBL-producing strains in diverse environments beyond clinical settings, such as in food sources.

The aim of this study was to determine the frequency of widespread expression of beta-lactamases (ESBLs) in clinical samples from patients in clinical settings and in non-clinical samples derived from natural resources, such as water bodies, soil structure and animal populations. By examining a variety of clinical and non-clinical samples, this study aims to provide an analysis of the antibiotic resistance landscape in the Tabuk region. 

The results of this study promise not only to deepen the global understanding of antibiotic resistance patterns but also to guide local public health interventions and tailored antimicrobial stewardship programs to reduce the spread of resistant bacteria in Tabuk and beyond [3]. This research will provide novel insights into the prevalence of ESBL-producing bacteria in Tabuk, Saudi Arabia, across clinical and environmental settings. By analysing antibiotic resistance patterns, potential sources, transmission routes, and genetic markers, the study will offer a comprehensive view of the local antibiotic resistance landscape. These findings will contribute to global understanding while specifically informing targeted interventions and stewardship programs for the Tabuk region, ultimately aiding in the broader fight against antibiotic resistance.

## 2. Materials and Methods

The current prospective study was conducted in the Department of Medical Microbiology, Faculty of Medicine, in collaboration with the Department of Mathematics, Faculty of Science, at the University of Tabuk, Tabuk, KSA, from December 2023 to February 2024. Prior to its commencement, the study obtained the requisite ethical approval from the Research Ethics Committee (REC) of the University of Tabuk.

### 2.1. Specimens

**Figure 1:**
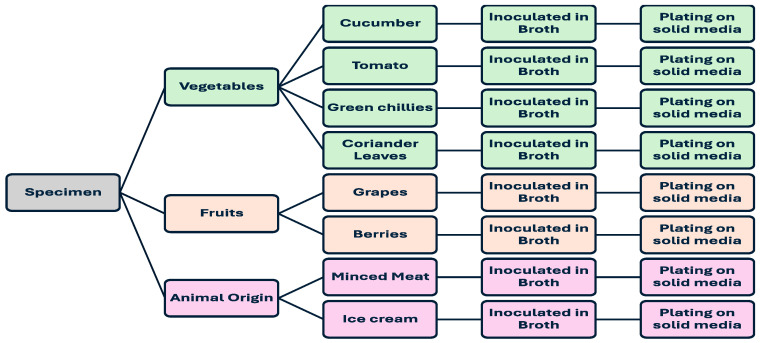
A diagram displaying the different food samples used in this study along with a summary of their processing steps.

Figure 1 illustrates the comprehensive steps delineating the various food-borne specimens utilised in this study along with their respective processing steps. All food items were meticulously gathered in sterile polythene bags and swiftly transported to the laboratory for subsequent processing.

### 2.2. Specimen Collection and Processing

To assess bacterial contamination, sterile swabs were used to collect samples from various food items. For most items, a 5.0 cm × 2.5 cm area was swabbed, while for coriander and mint leaves, a 2.5 cm × 2.5 cm area was swabbed on both sides. These swabs were then cultured on blood agar (BA) and McConkey agar (McA) plates and incubated overnight. Minced meat, chopped meat, ice cream, and Jalebi were processed using the same methods. All identified Gram-negative bacteria were further characterised to the species level using various biochemical tests, including catalase, oxidase, motility, coagulase, carbohydrate fermentation, and other standardised methods [4].

### 2.3. Clinical Specimen Collection and Processing

Thirty-six pre-characterised strains were collected from microbiology laboratory of the government hospital in Tabuk City. The Kirby Bauer disc diffusion method was used for a susceptibility test, as per the Clinical and Laboratory Standards Institute [5] guidelines on Mueller–Hinton agar. Amikacin (30 µg), ceftazidime (30 µg), cefepime (30 μg), levofloxacin (5 μg), piperacillin (100 μg), imipenem (10 μg), cefotaxime (30 µg), cefotaxime/clavulanic acid (30/10 µg), gentamicin (10 μg), cefoxitin (30 μg), ofloxacin (5 μg) and amoxicillin (30 μg) were used in this study [Hi-Media labs, India]. The results were interpreted in accordance with the manufacturer’s recommendations (Hi-Media labs, Mumbai, India).

### 2.4. Extended-Spectrum Beta-Lactamase (ESβL) 

#### 2.4.1. Screening for Potential ESBLs 

Bacteria were considered to be potential ESBL producers when they showed a ≤27 mm zone of inhibition with cefotaxime (30 μg) and ≤22 mm with ceftazidime (30 μg), as recommended by CLSI guidelines [6].

#### 2.4.2. Confirmation for ESBLs 

The ceftazidime (30 g), cefotaxime (30 g), cefepime (30 g), ceftazidime + clavulanic acid (30 g/10 g), cefotaxime + clavulanic acid (30 g/10 g), and cefepime + clavulanic acid (30 g/10 g) were placed at appropriate distances on the MHA plate and incubated as 37 °C for 18 h. A zone of inhibition difference of more than 5 mm in clavulanic acid in comparison with a single ceftazidime or cefotaxime disc was considered as being confirmed ESBL positive [6]. The control strains used in this study were *E. coli* ATCC 25,922 (non ESBL-producer) and *Klebsiella pneumoniae* 700,603 (ESBL-producer).

#### 2.4.3. Preparation of DNA Template

Thirteen phenotypic ESBL positive strains of clinical origin were selected to form our molecular study. We did not select strains from the non-clinical setting as none of the isolates showed ESBL positivity in the phenotypic test. A template from bacteria was prepared from freshly cultured bacterial isolates by suspending 3–5 colonies in 50 µL of molecular grade water and then heating at 95 °C for 5 min before immediately chilling at 4 °C. Positive controls harbouring *bla_CTX-M_, bla_TEM_, bla_SHV_* and *bla_AmpC_* and negative control (*E. coli* ATCC 25922) were processed in the same way for DNA extraction [7].

#### 2.4.4. Detection of Bla Genes by PCR

Molecular detection of *bla_CTX-M_*, *bla_TEM_*, *bla_SHV_* and *bla_AmpC_* genes in all food and clinical isolates was carried out utilising polymerase chain reactions (PCRs) by following previously described methods with slight modifications [2,8]. The specific primers used for the detection of these genes are listed in Table 1. A polymerase chain reaction (PCR) was used to detect the presence of the following four antibiotic resistance genes: *bla_TEM_, bla_SHV_, bla_AmpC_,* and *bla_CTX-M_*. For *bla_TEM_, bla_SHV_,* and *bla_AmpC_*, the multiplex PCR cycling conditions included an initial denaturation step at 95 °C for 15 min followed by 35 cycles of 94 °C for one minute (denaturation), 58 °C for two minutes (annealing) and 72 °C for three minutes (extension). The final step involved an elongation phase at 72 °C for 10 min. 

For blaCTX-M detection, the PCR cycling conditions were slightly different, as follows: an initial denaturation at 94 °C for seven minutes followed by 35 cycles of 94 °C for 50 s (denaturation), 50 °C for 40 s (annealing) and 72 °C for one minute (extension). The final elongation step was at 72 °C for five minutes. The PCR products were then analysed by gel electrophoresis, with 5 µL of each product being loaded onto a 2% agarose gel. Control samples containing known *bla_CTX-M_, bla_TEM_, bla_SHV_*, and *bla_AmpC_* sequences were used as molecular weight markers [7].

## 3. Results

### 3.1. Bacterial Isolates Obtained from Food Specimens

A total of 21 bacterial isolates were recovered from food samples. These isolates comprised seven *Klebsiella pneumoniae*, eight *E. coli*, 4 *Pseudomonas* sp., one *Citrobacter koseri*, and one *Acinetobacter* sp. A detailed breakdown of the isolates from each food type is presented in Table 2.

### 3.2. Bacterial Isolates Obtained from Clinical Specimens

Overall, 36 bacterial strains were collected from various hospitals in the city of Tabuk, KSA. The most frequently found clinical isolates were *E. coli* and *Pseudomonas* sp., both representing 22.2%, followed by *Proteus mirabilis* (13.9%). Detailed tabulated results are depicted in Table 2.

### 3.3. Antibiotic Resistance Pattern

#### 3.3.1. Isolates from Food Specimens

Table 3 details the antibiotic resistance profiles of the food-borne bacterial isolates. Gentamicin exhibited the highest resistance rate (19.0%), followed by amoxicillin (14.3%). Ceftazidime, cefepime, cefotaxime, ofloxacin, and cefoxitin showed similar resistance rates of 9.5% each. Notably, all isolates remained susceptible to imipenem. 

#### 3.3.2. Isolates from Clinical Specimens

Clinical isolates exhibited the highest resistance rates against third-generation cephalosporins. Cefotaxime showed the greatest resistance (88.9%), followed by ceftazidime (80.6%), cefepime (63.9%) and cefoxitin (58.3%). Amikacin and levofloxacin also displayed significant resistance levels at 61.1% and 52.8%, respectively. Resistance to amikacin, levofloxacin and amikacin was noticed in 61.1%, 52.8% and 50.0% isolates, respectively, and only two isolates were resistant to imipenem (Table 3).

### 3.4. The Magnitude of ESBL Detection 

#### 3.4.1. In Clinical Samples 

In an ESBL screening test using the disc diffusion method, 58.3% of all isolates tested positive. The highest and least positive intraspecies for screening ESBLs were *E. coli* (25.0%) and *Proteus* sp. (3.1%) (Table 4). In the combination disc method (confirmatory), 36.11% were found to be positive. The highest and the least intraspecies ESBL positive specimens were identified as *E. coli* (21.9%) and *Klebsiella oxytoca* (3.1%). There was a 38.07% reduction in ESBL positivity isolates in total from the phenotypic to confirmatory test results.

#### 3.4.2. In Food Specimens

Of all the isolates, 9.5% were found to be positive in the screenings for ESBL using the disc diffusion method. The phenotypic ESBL producers varied among isolated organisms. Only single isolates of *Klebsiella pneumoniae* and *E. coli* were to be found to be positive during the initial screening, and none of the isolates were found to be positive during the confirmatory test.

### 3.5. Occurrence of Bla Genes

**Figure 2:**
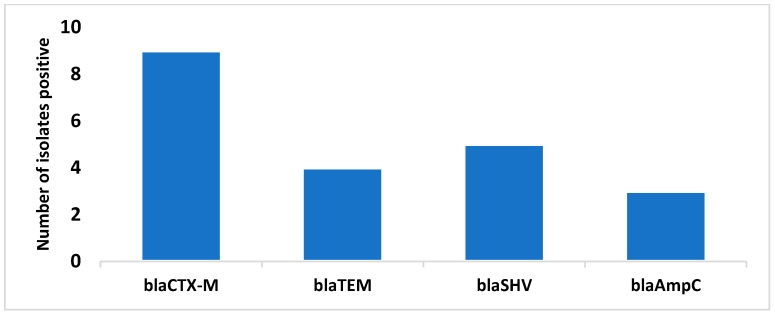
*Bla* ESBL gene positivity.

The prevalence of *bla* genes (*bla_CTX-M_*, *bla_TEM_*, *bla_SHV_,* and *bla_AmpC_*) in clinical isolates is illustrated in Figure 2. Among the isolates, *bla_CTX-M_* was the most common ESBL, found in nine isolates (69.2%), followed by *bla_SHV_* in five isolates (38.4%), *bla_TEM_* in four isolates (30.7%), and *bla_AmpC_* in three isolates (23.0%). Interestingly, none of the ESBL genes were detected in food-borne bacterial isolates. This high prevalence of *bla_CTX-M_* genes in clinical isolates and their potential for horizontal gene transfer indicate a significant challenge in antibiotic resistance. These genes contribute to ESBL production, making bacteria resistant to third-generation cephalosporins and posing challenges to antimicrobial therapy. Without proper control measures and antibiotic stewardship programs, the dissemination of *bla_CTX-M_* genes could lead to a global spread of multidrug-resistant bacteria, complicating treatment options and increasing the risk of infections that are difficult to manage.

**Figure 3:**
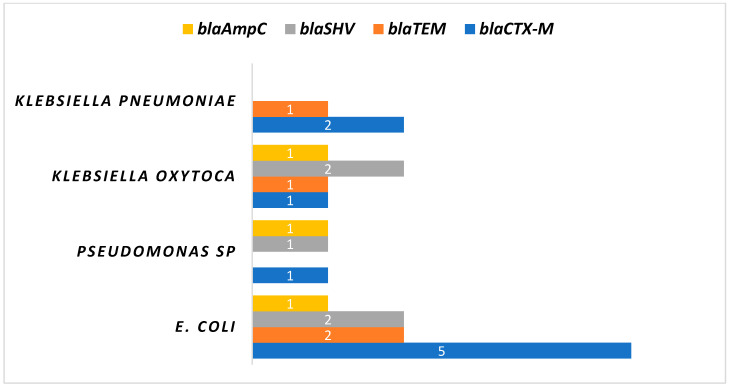
*Bla* ESBL gene positive results per organism.

**Figure 4:**
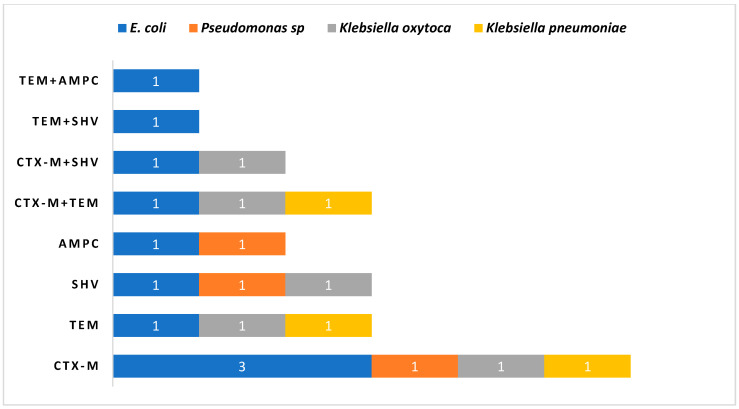
Clinical isolates distribution pattern of mono and two *bla_ESBL_* gene pattern analysis.

Upon genotype distribution analysis in Figure 3, it was observed that most strains harboured a single gene. Specifically, six strains carried *bla_CTX-M_* alone, while three strains each contained *bla_SHV_* and *bla_TEM_* genes and two strains possessed the *bla_AmpC_* gene. Furthermore, a combination of two *bla* genes was observed, with *bla_CTX-M_ + bla_TEM_* present in three isolates, *bla_CTX-M_ + bla_SHV_* in two isolates, and *bla_TEM_ + bla_SHV_* as well as *bla_TEM_ + bla_AmpC_* each found in one strain. The distribution of ESBL genes based on organisms is depicted in Figure 4.

## 4. Discussion

The phenomenon of antibiotic resistance is the result of various factors, including the overuse and misuse of antibiotics in both human medicine and agriculture, leading to the development of resistant bacterial strains [9]. ESBL-producing bacteria are a major concern in clinical settings because they limit treatment options for infections and are associated with higher morbidity and mortality rates [10]. The study of antibiotic resistance and extended-spectrum ß-lactamase (ESBL) production in clinical and non-clinical isolates in Tabuk is an essential investigation that aims to understand the prevalence and mechanisms of resistance among bacterial populations in this region. 

A total of 57 (36 from hospital sources and 21 from non-clinical sources) different types of isolates were analysed in this study. Among the clinical and non-clinical isolates, the most prevalent were *E. coli* and *Klebsiella pneumoniae* respectively. In the Tabuk region, localised studies are vital in gathering data on the specific strains of bacteria that are present, their resistance profiles, and the factors contributing to their spread. This information is crucial for the development of targeted infection control interventions and antibiotic stewardship programs. Local epidemiological data can aid healthcare providers in selecting the most appropriate antibiotics for treatment, thus combating the spread of resistance more effectively. Most of the clinical isolates were resistant to the tested antibiotics compared to the isolates of a non-clinical origin (Supplementary Tables S1 and S2). The analysis of food specimens revealed the presence of 21 bacterial isolates, which were categorised as follows: 7 isolates of *Klebsiella pneumoniae*, 8 isolates of *E. coli*, 4 isolates of *Pseudomonas* species (sp) and single isolates of *Citrobacter koseri* and *Acinetobacter* sp. (Table 2). The preponderance of *E. coli* (38%) and *Klebsiella pneumoniae* (33%) underscores their significant role in foodborne diseases. According to Beuchat and Ryu [11], such pathogens are critical indicators of food safety, as they often originate from fecal contamination. *Klebsiella pneumoniae* and *E. coli* are notable for their ability to thrive in diverse environments, making them robust contaminants in the food supply chain [12]. *Pseudomonas* species were detected in 19% of the food specimens, emphasising their importance given their known resistance to various disinfection methods and their association with spoilage and human infections [13]. The detection of *Citrobacter koseri* and *Acinetobacter* sp., though lower in frequency (each at 4.8%), also warrants attention as both are associated with significant opportunistic infections, further stressing the necessity for stringent food safety controls.

This study also incorporates samples from animal-based foods and sweets openly sold in Saudi markets, along with a selection of clinical bacteria, to compare their resistance patterns and the presence of resistance genes. *E. coli* and *Klebsiella pneumoniae* were the most frequently isolated bacteria from the non-clinical samples in this study. Many of the food samples had mixed bacterial growth. It was discovered, frighteningly, that just running water over salad veggies would not get rid of microorganisms after washing the specimens. Therefore, it is recommended that antiseptic treatments, such as potassium permanganate, should be used to sterilise such vegetables. The patterns of antibiotic susceptibility varied significantly between clinical isolates and food-borne isolates. The isolates that were food-borne demonstrated the greatest gentamicin resistance (19%), followed by amikacin (14.3%). Table 3 indicates that a mere 9.5% of these isolates exhibited maximum resistance to any third-generation cephalosporins. On the other hand, most of the cephalosporin resistance (88.9%) was demonstrated by clinical isolates. Clinical isolates had resistance rates of 61.1%, 52.8% and 50.0% in regard to amikacin, levofloxacin, and piperacillin, respectively. Imipenem resistance was also present in two clinical isolates. 

A comprehensive examination of clinical specimens from hospitals in Tabuk, KSA, led to the identification of 36 bacterial strains. The dominant isolates were *E. coli* and *Pseudomonas* species, each making up 22.2% of the total specimens. The recurrent isolation of these bacteria from clinical settings is consistent with their known status as leading causes of nosocomial infections, particularly in intensive care units (ICUs) [14,15]. The high incidence of *Proteus mirabilis* (13.9%) further aligns with previous findings that spotlight this pathogen’s role in urinary tract infections and catheter-associated infections [16]. The overlapping presence of *E. coli* and *Pseudomonas aeruginosa* in both food and clinical specimens may signify potential cross-contamination pathways, highlighting the public health risk posed by antibiotic-resistant bacteria traversing from food sources to clinical environments and vice versa [17].

The resistance profiles of the bacterial isolates from food specimens provided important insights into the current state of antibiotic resistance. The highest resistance was observed against gentamicin (19%), a critical antibiotic in both human and veterinary medicine. This parallels global concerns about antibiotic use in agriculture contributing to the resistance issue [18]. Amoxicillin resistance was noted at 14.3%, a worrying trend given that amoxicillin is a commonly prescribed antibiotic. Resistance to cephalosporins (ceftazidime, cefepime, cefotaxime, cefoxitin), as well as ofloxacin, were noted at 9.5% each. Interestingly, no resistance was detected against imipenem in the foodborne isolates, underscoring the continued efficacy of carbapenems against these pathogens. This finding aligns with studies by Nordmann and Poirel [19], indicating that carbapenems remain largely effective against many resistant bacterial strains, though vigilance is necessary to prevent the emergence of resistance.

The antibiotic resistance pattern among clinical isolates was particularly concerning, with high resistance observed against third-generation cephalosporins as follows: cefotaxime (88.9%), ceftazidime (80.6%), cefepime (63.9%), and cefoxitin (58.3%). These rates suggest a significant presence of extended-spectrum-beta-lactamase (ESBL)-producing organisms, which complicate treatment protocols due to their ability to hydrolyse these antibiotics [20]. Moreover, resistance to amikacin (61.1%) and levofloxacin (52.8%) was also notable. The similar resistance rates against these two antibiotics emphasise the adaptations these bacteria have undergone, likely driven by selective pressure from antibiotic use in clinical settings [21]. Despite the high resistance rates to multiple antibiotics, resistance to imipenem was minimal, with only two isolates showing resistance. This observation supports the strategic use of carbapenems as a last resort for multidrug-resistant infections while underscoring the need for judicious use to preserve their effectiveness [22].

Since antibiotics are widely accessible over the counter in the world, the irrational use of antibiotics is blamed for the high incidence of these resistance genes [23]. The bacterial population linked with plants may freely spread these resistance genes when sewage water is used for irrigation, a typical irrigation technique in developing countries. Considering these details, we designed this investigation to find out how common enteric infections are in vegetation and to look for the presence of *bla* genes. 

In clinical settings, it was observed that 58.3% of isolates tested positive for ESBL using the disc diffusion method, indicating a significant presence. Among these, *E. coli* exhibited the highest positivity rate at 25.0%, while the Proteus species showed the lowest rate at 3.1%. This variation highlights the differential distribution of ESBL producers among bacterial species. A recent study from Tabuk also reported a high prevalence of ESBL-producing *E. coli* in clinical samples, emphasising the critical role of this pathogen in local hospitals [24]. Furthermore, the confirmatory combination disc method showed a reduction in positivity to 36.11%, with *E. coli* again being the predominant ESBL producer at 21.9%, followed by *Klebsiella oxytoca* at 3.1%. This represents a notable reduction of 38.07% from screening to confirmatory tests.

This pattern is corroborated by a study from Saudi Arabian hospitals, where a marked reduction in ESBL positivity was observed from initial screening to confirmatory testing [25]. ESBLs are produced through different Gram-negative rod-shaped bacteria. In this regard, ESBLs are made through *K. pneumoniae*, the ESBL-producing organism. There has been an instant elevating occurrence of ESBL production among cephalosporins due to the excessive use and misuse of broad-spectrum antibiotics among ESBL enzymes are essentially plasmid mediated. The consistent predominance of *E. coli* as an ESBL producer in clinical samples aligns with local epidemiological data, underscoring the pathogen’s critical role in the dissemination of antibiotic resistance. The same plasmid can also confer additional resistance contributors to aminoglycosides and fluoroquinolones. In addition, most ESBL isolates demonstrate cross-resistance to non-β-lactam antibiotics, including nitrofurantoin, TMP-SMX, ciprofloxacin and aminoglycosides, stressing additional therapeutic challenges to both clinical microbiologists and clinicians. According to most of the reports, delays in opposite therapy and an associated elevation in the rates are caused by such enzymes [26].

The study reports a lower rate of ESBL positivity in food specimens, with 9.5% screening positive via the disc diffusion method. Only single isolates of *Klebsiella pneumoniae* and *E. coli* were identified as phenotypic ESBL producers; however, none tested positive in confirmatory tests, indicating a 100% reduction in ESBL positivity. This stark contrast suggests a lesser extent of ESBL contamination in food sources compared to clinical environments. Research in Saudi Arabia highlights the emerging concern of ESBLs in foodborne pathogens, although reported rates vary. For instance, a study detected low prevalence but did acknowledge the potential risk of food as a vehicle for ESBL-producing bacteria [27]. The negligible detection in confirmatory tests could reflect insufficient bacterial load or the high sensitivity and specificity of the confirmatory methods that were employed.

The most common ESBL gene in the present study has been found to be *bla_CTX-M_*, followed by *bla_SHV_*. The phenotypic demonstration of ESBL synthesis between food and clinical isolates showed a significant difference. There was a 38% reduction in the clinical samples in the phenotypic ESBL confirmatory test in comparison to the screening and a 100% reduction in non-clinical samples. It is interesting to note that, whereas *bla_CTX-M_*, *bla_TEM_*, *bla_SHV_,* and *bla_AmpC_* genes were detected in 69.2%, 30.7%, 38.4%, and 23.0% of clinical isolates, respectively, none of the food-borne isolates had any *bla* genes. In the Indian continent, CTX-M were the most common and broadly disseminated genes in the clinical bacterial population [28,29]. A study conducted by [30] shows the presence of *bla_CTX-M_*, bla*_TEM_* and *bla_SHV_* genes in clinical as well as non-clinical *E. coli*. Even though these resistance genes have not yet been discovered in food vegetation, there is still a serious risk in regard to their worldwide dissemination. This study emphasises how important it is to put in place stringent irrigation and antibiotic usage regulations to stop the spread of these resistant genes. Furthermore, it is imperative to stop antibiotics from being sold over the counter in developing countries. Genotypic analyses revealed that most strains harboured a single type of *bla* gene, with *bla_CTX-M_* being observed alone in six strains, followed by *bla_SHV_* and *bla_TEM_* in three strains each and *bla_AmpC_* in two strains. The presence of multiple *bla* genes, such as *bla_CTX-M_ + bla_TEM_*, further illustrates the genetic diversity and potential for horizontal gene transfer among these pathogens. 

Saudi Arabian studies corroborate these findings, identifying *bla_CTX-M_* as the predominant genotype in clinical isolates from previous studies that have provided similar insights, confirming the high prevalence and genetic diversity of *bla* genes in the region [24,31,32]. The combination of *bla* genes in single strains signifies the complex nature of ESBL-mediated resistance, posing challenges to antimicrobial therapy. To the greatest extent of our knowledge, this is the first report from the Tabuk region looking at these resistance genes in populations of foodborne bacteria and one of the first studies exploring the presence of *bla* genes, including *bla_CTX-M_*, within Tabuk clinical isolates. The limitations of this study as food sources may not represent the full extent of antibiotic resistance in other environments, and the lack of gene sequencing limits the understanding of specific variants and their evolution. Moreover, the single time point study prevents the assessment of temporal trends in antibiotic resistance. Finally, the study does not delve into the molecular mechanisms underlying resistance, hindering a comprehensive understanding beyond ESBL production. 

## 5. Conclusions

The study highlights the critical issue of antibiotic resistance and ESBL production in Tabuk, demonstrating significant resistance among clinical isolates. The high prevalence of resistance to third-generation cephalosporins in clinical settings underscores the urgency of enhancing infection control measures and implementing robust antibiotic stewardship programs. In contrast, foodborne isolates showed lower rates of resistance and ESBL production, but the detection of resistance genes like *bla* genes calls for stringent food safety practices and proper antibiotic use in agriculture. The presence of multiple *bla* genes in single clinical strains indicates the complex nature of resistance and the potential for horizontal gene transfer. The study stresses the importance of multicentric data acquisition in formulating effective treatment guidelines and preventive strategies to curb the spread of antibiotic-resistant bacteria in both healthcare and community settings.

## Figures and Tables

**Table 1:** The primers used and the expected amplicon sizes for PCR-based detection of the *bla_CTX-M_, bla_TEM_, bla_SHV_*, and *bla_AmpC_* genes.

Targets	Primer Sequence (5′ to 3′)	Amplicon Size (bp)
*bla_CTX-M_*	F-ATGTGCAGYACCAGTAARGT	593
	R-TGGGTRAARTARGTSACCAGA	
*bla_TEM_*	F-KACAATAACCCTGRTAAATGC	936
	R-AGTATATATGAGTAAACTTGG	
*bla_SHV_*	F-TTTATCGGCCYTCACTCAAGG	930
	R-GCTGCGGGCCGGATAACG	
*bla_AmpC_*	F-CCCCGCTTATAGAGCAACAA	634
	R-TCAATGGTCGACTTCACACC	

**Table 2:** Details of Clinical and Non-Clinical Isolates.

Clinical Isolates	N	%	Non-Clinical Isolates	N	%
*E. coli*	8	22.2	*Klebsiella pneumoniae*	7	33.3
*Pseudomonas* sp.	8	22.2	*E. coli*	8	38.1
*Klebsiella oxytoca*	4	11.1	*Pseudomonas* sp.	4	19.0
*Klebsiella pneumoniae*	3	8.3	*Citrobacter koseri*	1	4.8
*Proteus vulgaris*	4	11.1	*Acinetobacter* sp.	1	4.8
*Proteus mirabilis*	5	13.9			
*Morganella morganii*	1	2.8			
*Citrobacter* sp.	3	8.3			

**Table 3:** Antibiotic resistance pattern of Clinical and Non-Clinical isolates.

Antibiotic Resistance Profile	Clinical [N (%)]	Non-Clinical [N (%)]
Amikacin	22 (61.1)	1 (4.8)
Ceftazidime	29 (80.6)	2 (9.5)
Cefepime	23 (63.9)	2 (9.5)
Levofloxacin	19 (52.8)	1 (4.8)
Piperacillin	18 (50.0)	1 (4.8)
Cefotaxime	32 (88.9)	2 (9.5)
Ofloxacin	16 (44.4)	2 (9.5)
Imipenem	2 (5.6)	0 (0.0)
Cefoxitin	21 (58.3)	2 (9.5)
Gentamicin	14 (38.9)	4 (19.0)
Amoxicillin	16 (44.4)	3 (14.3)

**Table 4:** ESBL pattern of clinical and non-clinical isolates (organism wise details).

Clinical	Phenotypic ESBL N (%)	Confirmatory ESBL N (%)
*E. coli*	8 (25.0)	7 (21.9)
*Pseudomonas* sp.	5 (15.6)	3 (9.4)
*Klebsiella oxytoca*	3 (9.4)	1 (3.1)
*Klebsiella pneumoniae*	3 (9.4)	2 (6.3)
*Proteius vulgaris*	1 (3.1)	0 (0.0)
*Proteus mirabilis*	1 (3.1)	0 (0.0)
*Morganella morganii*	0 (0.0)	0 (0.0)
*Citrobacter* sp.	0 (0.0)	0 (0.0)
**Non-Clinical**		
*Klebsiella pneumoniae*	1 (50)	0 (0)
*E. coli*	1 (50)	0 (0)
*Pseudomonas* sp.	0 (0)	0 (0)
*Citrobacter koseri*	0 (0)	0 (0)
*Acinetobacter* sp.	0 (0)	0 (0)

## Data Availability

The raw data of all analyses are available from the corresponding author (M.Z.) upon request.

## Supplementary Materials

The following supporting information can be downloaded at https://www.mdpi.com/article/10.3390/medsci12030042/s1: Table S1: Antibiotic Resistance Pattern (Organism Wise Details) of Clinical Isolates; Table S2: Antibiotic Resistance Pattern (Organism Wise Details) of Non-Clinical Isolates.
