# Supplementary material for: Antibiotic Resistance & Extended-Spectrum ß-Lactamase Production in Clinical and Non-Clinical Isolates in Tabuk
Source: Med Sci (Basel). 2024 Aug 18;12(3):42. doi: 10.3390/medsci12030042 (PMC11348262; doi:10.3390/medsci12030042)
Supplement: Supplementary file 1 [file medsci-12-00042-s001.zip › medsci-3149760-supplementary.pdf]

medical sciences

MDPI

Table S1. Antibiotic Resistance pattern (organism wise details) of clinical isolates.

Antibiotic Resistance profile	*E. coli*	*Pseudomonas sp*	*Klebsiella oxytoca*	*Klebsiella pneumoniae*	*Proteius vulgaris*	*Proteus mirabilis*	*Morganella morganii*	*Citrobacter sp*
Amikacin	*6*	4	2	2	2	3	1	2
Ceftazidime	*7*	7	3	2	3	4	1	2
Cefepime	*6*	4	2	2	3	4	1	1
Levofloxacin	*7*	4	1	2	1	3	0	1
Piperacillin	*6*	1	2	1	2	4	1	1
Cefotaxime	*8*	8	4	2	3	4	1	2
Ofloxacin	*5*	4	3	1	1	1	1	0
Imipenem	*0*	2	0	0	0	0	0	0
Cefoxitin	*5*	4	3	1	2	4	1	1
Gentamycin	*5*	3	1	1	1	1	1	1
Amoxicillin	*5*	4	2	1	1	2	0	1

Data were presented as n as number, and unless otherwise indicated. Number of isolates resistance to antibiotic from respective organism [*E. coli*=8, *Pseudomonas sp*=8, *Klebsiella oxytoca*=4, *Klebsiella pneumoniae*=3, *Proteus vulgaris*=4, *Proteus mirabilis*=5, *Morganella morganii*=1, *Citrobacter sp*=3].

Table S2. Antibiotic Resistance pattern (organism wise details) of non-clinical isolates.

Antibiotic Resistance profile	*E. coli*	*Pseudomonas sp*	*Acinetobacter*	*Klebsiella pneumoniae*	*Citrobacter sp*
Amikacin	*1*	0	0	0	0
Ceftazidime	*1*	1	0	0	0
Cefepime	*1*	0	0	1	0
Levofloxacin	*1*	*ND*	*ND*	*ND*	*ND*
Piperacillin	*1*	*ND*	*ND*	*ND*	*ND*
Cefotaxime	*1*	1	0	0	0
Ofloxacin	*1*	0	1	0	0
Imipenem	*0*	0	0	0	0
Cefoxitin	*1*	0	1	0	0
Gentamycin	*2*	1	0	1	0
Amoxicillin	*1*	1	0	0	1

Data were presented as n as number, and unless otherwise indicated. Number of isolates resistance to antibiotic from respective organism [*E. coli*=8, *Pseudomonas sp*=4, *Acinetobacter sp*=1, *Klebsiella pneumoniae*=7, *Citrobacter sp*=1]. ND=not done.
